# Effect of Age on Physical Function Improvement in Older Veterans Enrolled in the Gerofit Exercise Program

**DOI:** 10.3390/healthcare14193348

**Published:** 2026-10-08

**Authors:** Angela J. Guo, Stacy S. Wilkins, Rebecca J. Melrose, Daniel Fernandez, Steven C. Castle, Katherine S. Hall, Cathy C. Lee

**Affiliations:** 1 Geriatric Research, Education, and Clinical Center and Mental Health, VA Greater Los Angeles Healthcare System, Los Angeles, CA 90073, USA; ajzeng@usc.edu (A.J.G.); stacy.wilkins@va.gov (S.S.W.); rebecca.melrose@va.gov (R.J.M.); daniel.fernandez@burrell.edu (D.F.);; 2 David Geffen School of Medicine at UCLA, Geffen Hall, 885 Tiverton Drive, Los Angeles, CA 90095, USA; 3 Geriatric Research, Education, and Clinical Center (GRECC) and Center of Innovation to Accelerate Discovery and Practice Transformation (ADAPT), Durham VA Health System, Durham, NC 27705, USA; 4 Roybal Center, Duke University, Durham, NC 27708, USA; 5 Center for the Study of Aging and Human Development, Duke University School of Medicine, Durham, NC 27710, USA

**Keywords:** physical activity, physical function, exercise, lower extremity strength, aging, older adults, oldest old, nonagenarian, Veterans

## Abstract

**Highlights:**

**What are the main findings?**

- Three-month participation in a supervised exercise program was associated with significant lower extremity strength gains across all age groups of community-dwelling older adults, including adults aged 90–99 years.

**What are the implications of the main findings?**

- Advanced age alone should not be considered a barrier to participation in exercise programs. Exercise can improve strength even among adults aged 90 years and older.

**Abstract:**

Background/Objectives: Aging is associated with declines in strength, balance, and mobility that contribute to falls, disability, and loss of independence. While exercise interventions improve physical function, limited data exist on community-dwelling adults aged 90 years and older. The purpose of this study was to evaluate the effect of age on physical function improvement following participation in an exercise program for older Veterans. Methods: We analyzed baseline and 3-month follow-up physical function data from 62 community-dwelling Veterans aged 60–99 years enrolled in the Veterans Affairs Gerofit, a supervised, individualized exercise program incorporating aerobic, resistance, balance, and flexibility training. Outcomes included lower extremity strength (30-s chair stands), mobility and balance (8-foot up-and-go), and cardiovascular endurance (6-min walk). Participants were grouped by age (60–69, 70–79, 80–89, 90–99). Paired samples *t*-tests assessed changes over time, and repeated-measures general linear models examined the effect of age on physical function improvement. Results: Across all participants, significant improvements were observed in chair stand and 8-foot up-and-go performance (both *p* < 0.001), whereas 6-min walk distance did not change significantly (*p* = 0.14). Chair stand performance improved in all age groups, including adults aged 90–99 (*p* < 0.01). Although baseline physical function declined with age, we did not detect differences in the rate of improvement in chair stand performance by age group (*p* = 0.69). Improvements in 8-foot up-and-go performance were observed in younger age groups and not in participants aged 90–99 years. Conclusions: Participation in a supervised exercise program was associated with significant improvements in lower extremity strength across all age groups, including adults aged 90–99 years. These findings support the inclusion of even the oldest adults in exercise programs to promote physical function.

## 1. Introduction

Aging is associated with progressive declines in muscle strength, balance, gait speed, and mobility, which contribute to increased risk of falls, disability, hospitalization, loss of independence, and mortality among older adults [1,2,3,4,5,6,7]. Measures of physical function, particularly lower extremity performance, are clinically important because they predict future disability, mobility, hospitalization, and mortality [8,9,10,11,12]. Preserving physical function is therefore a critical component of healthy aging and maintaining independence in later life.

Regular physical activity and structured exercise interventions have been shown to improve strength, mobility, functional independence, and quality of life in older adults while reducing frailty and fall risk [13,14,15,16,17]. Exercise interventions have demonstrated benefits across a variety of community and institutional settings. As a result, exercise is widely recommended to promote functional independence and healthy aging.

Although the benefits of exercise in older adults are well established, relatively little is known about the effects of exercise among adults aged 90 years and older. This population represents one of the fastest-growing age groups in the United States and is projected to increase substantially in the coming decades, highlighting the importance of identifying effective interventions that can preserve physical function and independence [18]. Existing studies involving older adults aged 90 years and older have primarily focused on nursing home or institutionalized populations and have reported mixed findings. Some studies have demonstrated significant strength gains in nonagenarians following resistance training, whereas others suggest that increasing age may be associated with reduced functional improvement [19,20]. Furthermore, few studies have examined community-dwelling older adults aged 90 years and older, and even fewer have compared exercise-related improvements across different age groups of older adults.

The Veterans Affairs (VA) Gerofit program is a supervised exercise program designed to improve health and physical function among older Veterans at risk for functional decline [21]. Using data from participants enrolled in the Greater Los Angeles VA Gerofit program, we examined changes in lower extremity strength, mobility and balance, and cardiovascular endurance over a 3-month period. Specifically, we compared outcomes across four age groups (60–69, 70–79, 80–89, and 90–99 years) to determine whether age influences physical function improvement secondary to exercise. We hypothesized that participants aged 90–99 years would demonstrate smaller improvements in physical function than younger age groups.

## 2. Materials and Methods

### 2.1. Study Design and Participants

This retrospective analysis used data collected from participants enrolled in the Greater Los Angeles VA Gerofit program between 2014 and 2020. The Gerofit program is a supervised exercise program designed to promote health and physical function among older Veterans over the age of 65 at risk for functional decline. Participants are referred to the program by their VA primary care providers. Exclusion criteria included: (1) inability to perform activities of daily living; (2) unstable angina; (3) proliferative diabetic retinopathy; (4) oxygen dependency; (5) incontinence; (6) open wounds; (7) no permanent residence; (8) need for VA transportation/travel; (9) cognitive impairment that impairs ability to follow instruction; and (10) history of disruptive behavior or behavior not conducive to group activity. Upon enrollment, participants completed a medical comorbidity survey and a baseline physical function assessment. Participants were given a personalized exercise prescription based on the results of the baseline physical function assessment and encouraged to attend supervised exercise sessions up to three times per week. Gerofit staff at the Greater Los Angeles VA received training in physical function assessment and exercise prescription from the primary Gerofit site at the Durham VA, followed by a reverse site visit to assess program fidelity. Exercise sessions were conducted in-person and included aerobic exercise, resistance training using resistance bands, free weights, resistance machines, and group exercise classes focusing on flexibility, mobility, and balance [21]. This study analyzed physical function assessment data at baseline and after 3 months. All data were collected for clinical purposes. The study was reviewed and approved by the Greater Los Angeles VA Institutional Review Board as a quality improvement/quality assurance project.

### 2.2. Physical Function Measures

Physical function was assessed using standardized measures from the Senior Fitness Test battery [22]. Lower extremity strength was assessed using the 30-s chair stand test, defined as the number of times a participant rises from a seated position and returns to sitting within 30 s. Mobility and dynamic balance were assessed using the 8-foot up-and-go test, which measures the time required to stand from seated position, walk 8 feet around a marker, and return to seated position. Cardiovascular endurance was assessed using the 6-min walk test, recorded as the total distance walked during 6 min. Assessments were completed at baseline and repeated approximately 3 months after enrollment.

### 2.3. Statistical Analysis

Age, sex, race/ethnicity, and self-reported medical comorbidities were obtained at baseline. Race/ethnicity was self-reported and participants were categorized as non-Hispanic White or racial/ethnic minority. The minority group included all participants who identified as Hispanic, Black, and/or Asian. A comorbidity index was calculated as the total number of comorbidities reported by the participant at the time of the baseline assessment. Analyses were conducted in SPSSv27. Participants were categorized into four age groups: 60–69, 70–79, 80–89, and 90–99 years. Data were assessed for normality by examining skewness/kurtosis and histogram plots. For measures showing skewness/kurtosis greater than |2|, nonparametric tests were used. Changes in physical function between baseline and the 3-month follow-up were evaluated using paired-samples *t*-tests or Wilcoxon signed ranks test. The 8-foot up-and-go data showed a positive skew and were log transformed for inferential analyses. To examine the impact of age on physical function outcomes, repeated-measures general linear models were performed with time (baseline vs. 3 months) as the within-subjects factor and age group as the between-subjects factor. No other variables were included in the model. Analyses were restricted to participants with complete baseline and 3-month data for the respective outcome. Additional exploratory analyses examined associations between age and comorbidity index. A one-way ANOVA was used to assess for differences in comorbidity index across age groups, and simple correlation was used to examine the association between comorbidity index and age.

## 3. Results

### 3.1. Participant Characteristics

Sixty-two participants completed baseline and 3-month follow-up assessments and were included in the analysis. Participants had a mean age of 80.1 ± 9.2 years (range 64–96), and 98% were male. Regarding race/ethnicity, 67.2% identified as non-Hispanic White, 3.3% as Hispanic White, 27.9% as non-Hispanic Black, 1.6% as non-Hispanic Asian. The mean comorbidity index was 8.73 ± 5.01. Participant characteristics by age group are presented in Table 1.

### 3.2. Physical Function Outcomes

Three participants had missing data for one or more physical function assessments (four missing assessment data points total), resulting in varying sample sizes across individual physical function tests.

Across all participants, significant improvements were observed in chair stands (n = 61, t(60) = −6.90, *p* < 0.001) and the 8-foot up-and-go (n = 62, t(61) = 4.15, *p* < 0.001) after 3 months of participation in Gerofit (Table 2). There was no significant change in 6-min walk distance (n = 59, t(58) = −1.76, *p* = 0.083).

### 3.3. Effect of Age on Physical Function Improvement

**Figure 1:**
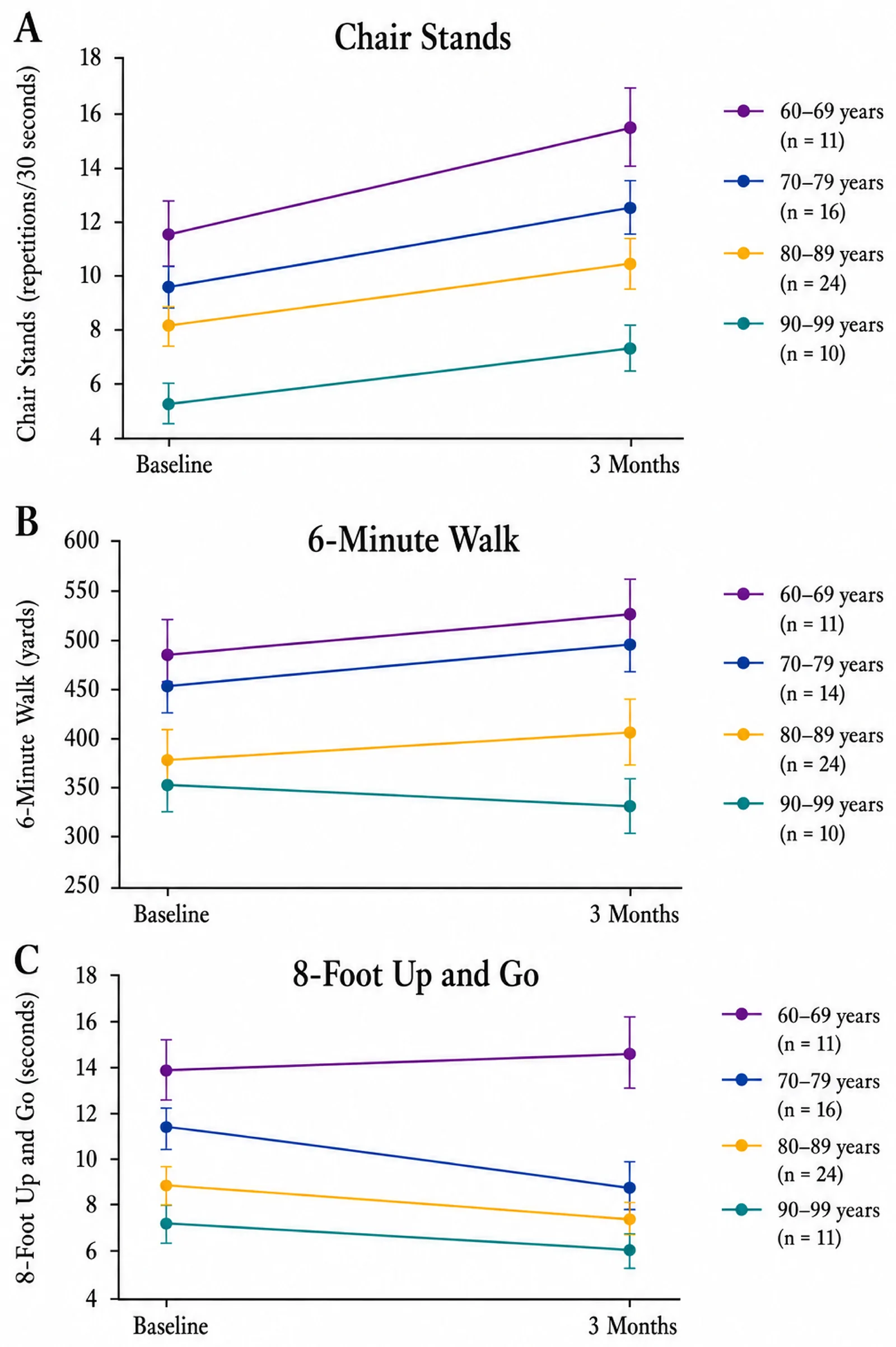
Physical function outcomes at baseline and 3-month follow-up by age group. Values represent mean ± SE (standard error). (**A**) Chair stand performance improved significantly in all age groups (all *p* < 0.05), with no Age × Time interaction (*p* = 0.69). (**B**) Performance in 6 min walk tests showed no significant effect of time (*p* = 0.21) and no Age × Time interaction (*p* = 0.14). (**C**) Performance in 8-foot up-and-go tests improved overall (*p* = 0.002), with a trend toward an Age × Time interaction (*p* = 0.05).

Age-related differences were examined using repeated-measures general linear models. Physical function trajectories from baseline to the 3 month follow-up by age group are shown in Figure 1.

For chair stand performance, there were significant effects of time (F(1, 57) = 43.96, *p* < 0.001, *η*^2^_p_ = 0.44) and age group (F(3, 57) = 5.13, *p* = 0.003, *η*^2^_p_ = 0.21), but no interaction between time and age group (F(3, 57) = 0.49, *p* = 0.69, *η*^2^_p_ = 0.03). Although older participants completed fewer chair stands overall, significant improvements were observed in all age groups, with estimated increases ranging from 2.17 to 3.36 repetitions (Table 3).

For the 6 min walk test, there was a significant effect of age group (F(3, 55) = 4.31, *p* = 0.008, *η*^2^_p_ = 0.19), with older participants walking shorter distances overall. However, there was no effect of time (F(1, 55) = 1.59, *p* = 0.21, *η*^2^_p_ = 0.03) and no interaction between time and age group (F(3, 55) = 1.89, *p* = 0.14, *η*^2^_p_ = 0.09).

For the 8-foot up and go test, there was a significant effect of time (F(1, 58) = 10.76, *p* = 0.002, *η*^2^_p_ = 0.16) and age group (F(3, 58) = 6.92, *p* < 0.001, *η*^2^_p_ = 0.26), as well as a trend towards an interaction between age group and time (F(3, 58) = 2.75, *p* = 0.05, *η*^2^_p_ = 0.12). Using both baseline and 3-month data, the 90–99 age group showed overall slowed performance relative to all other age groups (all *p*’s < 0.021).

Estimated changes and 95% confidence intervals by age group for all three physical function outcomes are presented in Table 3. For the 8-ft up-and-go, raw-scale values and changes are presented in Table 3 for clinical interpretability.

### 3.4. Comorbidities

Comorbidity data were available for 52 participants. There were no significant differences in comorbidity indices across age groups (F(3, 51) = 0.55, *p* > 0.05), and age was not correlated with comorbidity index (r = 0.009, *p* > 0.05).

## 4. Discussion

In this study of community-dwelling Veterans enrolled in the Gerofit exercise program, participation in a supervised exercise intervention was associated with significant improvements in lower extremity strength across all age groups and improvements in mobility and balance among some age groups. We did not detect significant differences in the rate of lower extremity strength improvement across age groups.

Age-related declines in strength, balance, and mobility are well documented and contribute to functional limitations and loss of independence among older adults [3]. Exercise interventions have consistently been shown to improve physical function, reduce functional decline, and reduce fall risk in older adults [15,23,24]. Recently, Manning et al. demonstrated that sustained participation in Gerofit attenuated age-related declines in physical function and that age did not affect physical function trajectories [25]. Our findings extend this work by demonstrating that even adults aged 90–99 years can achieve meaningful improvements in lower extremity strength over a relatively short 3-month period.

The most notable finding of our study was the improvement in chair stand performance across all age groups. Chair stand performance is a valid measure of lower body strength and is associated with muscle mass, disability risk, hospitalization, and mortality in older adults [8,26,27,28]. While participants aged 90–99 years performed fewer chair stands overall than younger participants, they demonstrated significant improvement in chair stand performance over the 3-month period. This finding expands on previous studies showing that the oldest old can benefit from resistance exercise and suggests that advanced age alone should not be considered a barrier to participation in exercise [19,29].

In contrast, improvements in mobility and balance were less consistent among the oldest participants. We observed a trend toward an interaction between age group and time for 8-foot up and go performance, with significant improvements observed only in the 70–79 and 80–89 age groups. Similarly, no significant changes were observed in 6 min walk performance in all age groups over the 3-month study period. The modest sample size, particularly in the 90–99 age group, may have limited statistical power to detect smaller changes in mobility, balance, and endurance outcomes. These findings may also indicate that strength adaptations occur earlier than improvements in more complex functional outcomes such as balance, mobility, and endurance. Consequently, longer intervention durations may be needed before improvements in these outcomes become apparent in the oldest adults. There is evidence that older adults may need to participate in exercise interventions for longer durations to benefit [30]. The absence of significant improvement in 6 min walk performance may also reflect characteristics of the intervention. Participants engaged in aerobic exercise used mostly the NuStep (Plymouth, MI, USA), which may not directly translate to walking performance.

Our findings are generally consistent with previous studies examining exercise interventions in the oldest adults. Fiatarone et al. reported significant strength gains following high-intensity resistance training in frail nursing home residents aged 87–96 years, while Serra-Rexach et al. demonstrated improvements in leg muscle strength following short-term exercise training in adults over the age of 90 [19,29]. Similarly, Cadore et al. reported improvements in strength, balance, and functional performance following a multicomponent exercise intervention in institutionalized nonagenarians [31]. Together with these studies, our findings suggest that meaningful functional adaptations remain possible even among adults in the tenth decade of life. Importantly, our study extends this literature by focusing on community-dwelling older adults and directly comparing exercise-related changes across multiple age groups.

This study has several strengths. First, the study included an ethnically diverse sample and a broad age range including a group of adults aged 90–99 years, a population that remains underrepresented in exercise intervention research. Second, our participants were community-dwelling older adults participating in a real-world clinical exercise program, enhancing the practical relevance of the findings. Third, the exercise program incorporated exercises that can easily be replicated by any older adult exercise program, increasing the applicability of the results to other clinical and community settings.

Limitations of our study include a small sample size, particularly for the 90–99 age group, which may have limited the ability to detect age-related differences in some outcomes. Additionally, this study did not include a nonexercise control group; therefore, regression to the mean, practice effects, or other temporal factors may have contributed to the observed changes in physical function. Since the analysis included participants with 3-month follow-up data, selection related to program retention may also have influenced the findings. Attendance frequency was not standardized across participants and individual attendance data were not available in the deidentified data set. Therefore, we could not determine whether exercise exposure differed systematically by age or adjust for differences in exercise exposure that may have influenced physical function outcomes. Furthermore, the comorbidity index did not account for disease type or severity; therefore, residual confounding by conditions affecting physical function cannot be excluded. In addition, Gerofit eligibility criteria selected for functionally independent older adults at risk of functional decline, which may limit generalizability to older adults with greater functional impairment or dependence in activities of daily living. Finally, our sample consisted almost entirely of male Veterans (98%), which may limit generalizability to older women and non-Veteran populations.

Future research should examine larger cohorts of community-dwelling older adults aged 90 years and older and evaluate the effects of different exercise modalities, intensities, and durations on physical function. Determining the optimal exercise prescription for adults over the age of 90 may help maximize physical function in this rapidly growing population. Recent international consensus recommendations support individualized multicomponent exercise programs, although evidence specific to adults aged 90 years and older remains limited [32].

## 5. Conclusions

In conclusion, participation in the Gerofit exercise program was associated with significant improvements in lower extremity strength across all age groups, including adults aged 90–99 years. We did not detect significant differences in the rate of strength improvement across age groups. These findings support the inclusion of the oldest adults in exercise programs. Ultimately, our findings reinforce the Gerofit motto: “You are never too old to get fit.”

## Figures and Tables

**Table 1:** Participant characteristics by age group.

Characteristic	60–69 Years (n = 11)	70–79 Years (n = 16)	80–89 Years (n = 24)	90–99 Years (n = 11)	Total (N = 62)
Male, n (%)	11 (100)	16 (100)	23 (95.8)	11 (100)	61 (98.4)
Minority race/ethnicity, n (%)	5 (45.5)	5 (31.3)	7 (30.4) *	3 (27.3)	20 (32.8)
Non-Hispanic White, n (%)	6 (54.5)	11 (68.7)	16 (69.6) *	8 (72.7)	41 (67.2)
Comorbidity index, mean ± SD	8.11 ± 5.33	8.46 ± 5.44	9.80 ± 4.03	7.50 ± 6.21	8.73 ± 5.01
Missing comorbidity data, n (%)	1 (9.1%)	3 (18.8%)	4 (16.7%)	1 (9.1%)	9 (14.5%)

* One participant in the 80–89 age group was missing race/ethnicity data. Abbreviation: SD, standard deviation.

**Table 2:** Changes in physical function outcomes following 3 months of participation in Gerofit.

Outcome	n	Baseline Mean ± SD	3-Month Mean ± SD	*p*-Value
Chair Stands (repetitions/30 s)	61	8.5 ± 4.2	11.0 ± 5.0	<0.001
6-Minute Walk (yards)	59	419.8 ± 158.0	433.6 ± 165.5	0.083
8-Foot Up-and-Go (seconds)	62	10.2 ± 5.9	9.1 ± 4.8	<0.001

Note: Sample sizes vary due to missing data for individual assessments. Abbreviation: SD, standard deviation.

**Table 3:** Estimated changes in physical function outcomes from baseline to 3-month follow-up by age group.

Outcome	Age Group	Estimated Change (3-Month–Baseline)	95% CI
Chair Stands (repetitions/ 30 s)	60–69	3.36	1.63 to 5.10
	70–79	2.63	1.19 to 4.06
	80–89	2.17	0.99 to 3.34
	90–99	2.20	0.38 to 4.02
6-Minute Walk (yards)	60–69	25.91	−9.87 to 61.68
	70–79	20.21	−11.50 to 51.93
	80–89	21.54	−2.68 to 45.76
	90–99	−26.50	−64.02 to 11.02
8-Foot Up and Go (seconds)	60–69	−0.50	−1.01 to 0.01
	70–79	−0.86	−1.67 to −0.05
	80–89	−2.13	−3.93 to −0.33
	90–99	0.15	−1.94 to 2.25

Note: Estimates and 95% CIs for chair stands and 6 min walk were derived from estimate marginal means from the repeated-measures general linear models. For 8-foot up-and-go, raw-scale mean changes and 95% CIs are presented for interpretability; inferential analyses were conducted using log-transformed values. Abbreviation: CI, confidence interval.

## Data Availability

The data presented in this study are available on request from the corresponding author because the data belong to the federal government and are restricted for Veteran privacy.

## Abbreviation

The following abbreviation is used in this manuscript:

VA	Veterans Affairs
